# Modelling psychiatric measures using Skew-Normal distributions

**DOI:** 10.1016/j.eurpsy.2010.08.006

**Published:** 2011-03

**Authors:** N. Counsell, M. Cortina-Borja, A. Lehtonen, A. Stein

**Affiliations:** a Department of Psychiatry, Warneford Hospital, University of Oxford, Oxford, OX3 7JX, UK; b Centre for Paediatric Epidemiology and Biostatistics, UCL Institute of Child Health, University College London, 30, Guilford Street, London, WC1N 1EH, UK

**Keywords:** Child Development, Biostatistics, Skewness

## Abstract

Data from psychiatric research frequently exhibit departures from Normality. Methods which utilise the data optimally to model the distribution directly are available. We highlight the issue of modelling skewness, resulting from screening instruments where the majority of respondents are healthy individuals and few participants have a value reflecting particular disorders.

## 1 Introduction

Variables arising from instruments designed to assess health status often follow asymmetric and long-tailed distributions, resulting from a majority of healthy individuals with low values and a few individuals with larger values reflecting particular disorders (e.g. screening questionnaires for symptoms and diagnoses). Although there may be other types of departure from Normality (e.g. value inflation, peakedness or flatness), skewness occurs frequently in a screening setting where the distributions are nearly Normal; thus it is particularly important to account for the values reflecting disorder whilst preserving the usual Normal properties of the general population. Non-Normally distributed responses are sometimes analysed using generalized linear models assuming a Poisson or a Gamma distribution. The former is adequate for count, not continuous, data and is often not flexible enough to cope with skewed data; the latter might be too different to the Normal for the purposes we have outlined. To adequately perform statistical analyses, we can rely on empirically chosen transformations (e.g. logarithmic, Box-Cox [4]) to make the data conform to the methods’ assumptions. However, it is not always possible to find a suitable transformation, and analysing data on a different scale might compromise interpretability. This means that rather than the dataset properties informing the statistical analyses, often inappropriate or non-optimal methods in which the data is not fully exploited are used, with assumption violations being accepted as an inevitable nuisance. This problem is compounded when analysing multivariate data. Although conclusions may not be compromised in large samples, distributional inferences from models including location, scale and shape parameters are preferable and aid our assessment of a population's characteristics. It is increasingly easy to implement and use statistical software to fit the models described below. In this paper, we highlight the utility of the bivariate Skew-Normal extension of the Normal distribution and apply it to a dataset from a clinical psychiatry research environment.

## 2 Methods

Non-parametric tests may be insufficient for a study's purpose, for instance, through the difficulties in fitting models when adjusting for covariates. In parametric modelling, data is often transformed, or the Normality assumption is inspected informally and any problem downplayed or ignored. Crawford et al. [5] have discussed this issue in a neuropsychological study, whilst Mardia [9] demonstrates the consequences of non-Normality and heterogeneity of variances in well-known multivariate statistical models. More recently, Elhai et al. [7] discuss these problems in the analysis of mental health services data and use zero-inflated distributions to overcome them for count data. Though these models are more flexible than the usual Normal models, they may not be applicable in analyses concerning multivariate, continuous, non-Normally distributed data. In this case, a more desirable solution, which is increasingly easy to implement, is to fit an appropriate model to the observed, asymmetric data.

Often it is important to analyse the joint distribution of multiple response variables, e.g. when modelling the same individuals measured at two or more time points or on a number of variables. In this case, deciding which transformations might be adequate to achieve the usual multivariate analysis of variance assumptions is difficult [12], as additional parameters are introduced to change the scale of the responses as required, which in turn complicate interpretability. Here we demonstrate the application of a bivariate Skew-Normal (SN_2_) model [3] to analyse measurements of maternal cognitions at two time points (3 and 6 months postnatal); a derived contour plot is useful to inform further extensions and gain insight in the bivariate data structure.

Data from the Oxford Parent Project, an ongoing longitudinal observational cohort study to investigate the influence of parenting on child development in the context of postnatal psychiatric disorder, is utilised. Three groups are included in the study: mothers with postnatal depression (*n *= 34), generalised anxiety disorder (GAD) (*n *= 52), and a healthy control group (*n *= 84).

There are three core components to psychological functioning: emotions, cognitions (thought processes) and behaviour. Most research in this area has concentrated on parental emotions and behaviour, with very little research on the thought processes that influence behaviour, especially when a parent is depressed or anxious. We are particularly interested in the core role of maternal cognitions, a key aspect of both depression and GAD. We term this process preoccupation, defined as a state of narrowed or self-focused attention in which one's mind is dominated by recurrent negative intrusive thoughts that are difficult to control or dismiss, and which recur due to their self-perpetuating nature [13]. Preoccupation was assessed using a 20-item questionnaire measuring the components of this cognitive process, with items scored from 0 to 4 (low-to-high) leading to total scores in the range 0 to 80 [10]. We investigate the relationship between preoccupation scores at two time points accounting for distributional properties of the data, and the study groupings.

The Normal distribution is particularly important in applied statistics and measurements of many real-world phenomena are well described by this probability model. However, due to some inherent biases in the underlying data-generating process, some measures will never be well characterised by the Normal distribution. The SN distribution [1] is a simple parametric generalisation of the Normal distribution which allows for model building, estimation and hypothesis testing. It contains the Normal model as a particular case and requires an additional shape parameter, *λ*, which governs skewness. This has been extended to the *k*-dimensional multivariate SN_*k*_ case [3] which, as well as parameters for location and scale, includes shape and dependence parameters.

We fitted SN_2_ models using the functions sn.mle and msn.mle from the package sn [2] in the statistical computing environment R version 2.7.1 [11] on a Windows^®^ platform. We used the Bayesian Information Criterion (BIC) as a goodness-of-fit criterion to rank competing models; [8] note that smaller values of BIC correspond to better fitting models.

## 3 Results

**Fig. 1:**
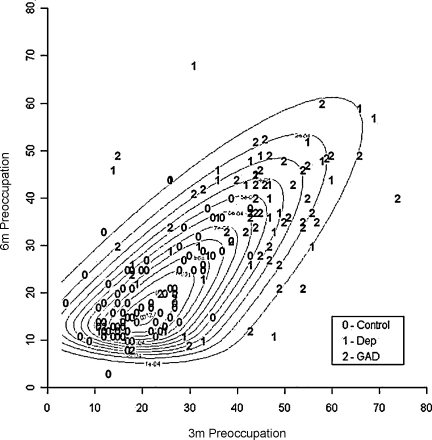
Contour plot of the bivariate Skew-Normal model for preoccupation scores at 3 and 6 months, by case group.

Plotting preoccupation scores from the two time points against each other (Fig. 1), a clear relationship is evident by case status; mothers with postnatal depression or GAD generally have high preoccupation at both time points, with more spread than controls. Higher values have more scope for change than lower values, while mothers with postnatal disorders may be more susceptible to higher levels of preoccupation than controls. Note that there are more individuals with high-to-low change than increasing scores. The data is bivariately skewed, with a large cluster in the bottom left graphical region the majority of whom are controls.

Maternal preoccupation scores have similar positively skewed shape coefficients corresponding to heavier right tails at both 3 and 6 months (*λ*_3m_ = 0.30, *λ*_6m_ = 0.31). Significant correlation exists between the two time points (Spearman's correlation = .711, *p* < .001), with median scores reducing from 31 to 28. The non-parametric Kruskal-Wallis *H* rank test shows that scores differ significantly in location among the three groups (*χ*^2^_3m_ = 86.3 on 2 d.f., *p* < .001; *χ*^2^_6m_ = 68.1 on 2 d.f., *p* < .001) with controls having lower levels of preoccupation than mothers with postnatal disorders. Although these tests do not require the Normality assumption, they are difficult to generalise to multivariate settings and to use allowing for covariates. Shapiro-Wilk tests confirm non-Normality (*p*'s < .001); logarithmic and Box-Cox transformations improve this situation, but not adequately as evidenced by the resulting highly curved QQ-plots and significant test statistics (data not shown). Thus, we use methods to account directly for this asymmetry; this is appropriate regardless of transformation success, and it could be argued that it is preferable to fit a model to the observed data rather than to adjust data to a given model.

The test for asymmetric departures from the bivariate Normal distribution, proposed by Mardia [9], yielded a significant result (*p* < .001). Fitting the SN_2_ model to compare the distributions of the three groups, whilst accounting for the joint asymmetry and dependence between preoccupation at 3 and 6 months, substantially improves the goodness-of-fit from the corresponding bivariate Normal model (BIC_N_ = 2674.8, BIC_SN_ = 2603.5). A univariate predictive regression model from this SN_2_ distribution could then be fitted to estimate missing 6 month preoccupation scores from 3 month data for example, considering the inclusion of case status or other covariates.

## 4 Discussion

Non-Normal models can help us understand asymmetric data arising from psychiatric research, further enabling appropriate analyses and inference depending on the study's aims, where alternative techniques are less flexible or overly complicated.

SN models can also be used to obtain confidence intervals based on parametric bootstrap methods [6] for functions of response variables whose distribution is difficult to characterise analytically. For example, bivariate centiles of preoccupation can be estimated from the fitted distribution, but the analytic form of their standard error is hard to obtain; however, they could be easily approximated using a parametric bootstrap procedure.

In this practical application of the SN_2_ distribution, we have demonstrated a method which was informed by the true data properties throughout. This allows for a modelling process when non-parametric tests are insufficient, with substantial scope for flexibility and complexity, which we believe could be used in many areas across behavioural and psychiatric research.

## Conflict of interest statement

None.
